# Microbial metabolite Urolithin A protects against inorganic arsenic-induced gut barrier dysfunction in humanized *AS3MT* mice

**DOI:** 10.1080/19490976.2026.2696618

**Published:** 2026-07-03

**Authors:** Sweta Ghosh, Zachary Matthew Vanwinkle, Kanchan Sinha Roy, Miroslav Stýblo, Mayukh Banerjee, James Collins, Venkatakrishna Rao Jala

**Affiliations:** a Department of Microbiology and Immunology, Brown Cancer Center, Center for Microbiomics, Inflammation and Pathogenicity, Center for Integrative Environmental Health Sciences, University of Louisville, Louisville, KY, USA; b Department of Nutrition, CB# 7461, Gillings School of Global Public Health, University of North Carolina at Chapel Hill, Chapel Hill, NC, USA; c Department of Pharmacology and Toxicology, University of Louisville School of Medicine, Louisville, KY, USA; d Center for Integrative Environmental Health Sciences, University of Louisville, Louisville, KY, USA; e Department of Microbiology and Immunology, Center for Microbiomics, Inflammation and Pathogenicity, University of Louisville, Louisville, KY, USA

**Keywords:** Gut microbiota, inorganic arsenic (iAs), gut barrier dysfunction, Urolithin A (UroA), hAS3MT transgenic mice, intestinal inflammation

## Abstract

Chronic exposure to inorganic arsenic (iAs) remains a major environmental health concern and is associated with significant gastrointestinal (GI) disorders, including gastroenteritis, diarrhea, and inflammatory bowel disease–like symptoms. Gut microbiota plays a critical role in mitigating arsenic toxicity, as germ-free or antibiotic-treated mice exhibit reduced fecal arsenic excretion and greater tissue accumulation. We previously showed that the microbial metabolite Urolithin A (UroA) protects against iAs-induced cytotoxicity, apoptosis, oxidative stress, and ROS production in vitro. In this study, using humanized *AS3MT* mice (mouse arsenic methyltransferase gene (*As3mt)*replaced with human *AS3MT,* h*AS3MT*), we evaluated the *in vivo* effects of iAs and UroA on gut barrier function. Long-term iAs exposure (100 ppb for 28 weeks) significantly reduced expression of tight junction proteins, indicating compromised intestinal barrier integrity. UroA treatment protected hAS3MT mice from iAs-induced gut permeability, inflammation, colon shortening, and elevated colon weight/length ratio. UroA also reduced iAs-induced inflammatory cytokines, myeloperoxidase (MPO) activity and preserved intestinal epithelial cell tight junction protein expression. Further, microbiome and metabolomic analysis suggested that UroA treatment protected from iAs-induced gut microbial dysbiosis, especially restored several beneficial bacterial strains and short chain fatty acids (e.g., acetate and butyrate) and led to gut homeostasis. Together, these findings demonstrate that UroA mitigates iAs-induced gut toxicity and restores microbiota homeostasis.

## Introduction

Humans are exposed daily to a wide range of environmental chemicals, including heavy metals, pesticides, organic pollutants, mycotoxins, and food additives.^1^ Among heavy metals and metalloids, arsenic, lead, mercury, cadmium, and chromium are well known for their adverse health effects.^2^ Approximately 60% of ingested metals are absorbed in the intestine, where they induce oxidative stress, disrupt gut barrier integrity, and promote intestinal inflammation.^3-5^ Chronic exposure to inorganic arsenic (iAs) has been strongly linked to gastrointestinal (GI) irritation, nausea, abdominal discomfort, vomiting,^6^ and significant alterations in gut microbiota.^7^ A notable example comes from Northport, Washington, where residents chronically exposed to iAs experienced 10–15 times higher incidence of inflammatory bowel disease (IBD) compared with the national average.^8^ Prior work, including our own, has shown that iAs damages the gut barrier, increases intestinal permeability, and promotes inflammation.^9-11^ Ingested iAs undergo extensive biotransformation by both gut microbiota and host cells, generating iAs species that influence toxicity and bioavailability.^12-15^ Growing evidence indicates that exposure to iAs disrupts intestinal homeostasis by inducing epithelial cell damage, compromising tight junction integrity, and altering mucus production, leading to deterioration of the gut barrier.^9-11^ Barrier dysfunction increases intestinal permeability, permitting translocation of microbial products such as lipopolysaccharide (LPS) across the epithelium and subsequent activation of inflammatory signaling. At the mechanistic level, iAs exposure promotes oxidative stress and mitochondrial dysfunction while activating pro-inflammatory pathways, including NF-κB and MAPK signaling, which drive the production of cytokines such as TNF-*α*, IL-6, and IL-1β.^16^

Recent studies demonstrate that gut microbiota is essential for protection against acute arsenic toxicity. Mice depleted of microbiota excrete less arsenic in stool and accumulate more in organs, while human microbiota transplantation restores protection.^15^ Gut microbiome analyzes of individuals exposed to iAs show that higher levels are associated with an increased abundance of pathogenic bacteria and a decreased abundance of beneficial commensal species.^17^ ^,^ ^18^ Human gut microbiota can chemically transform arsenic species, influencing their bioavailability and toxicity.^12^ One potential mechanism is the production of microbiota-derived metabolites possibly provide the protection against iAs-induced tissue injury.^13^ ^,^ ^14^ ^,^ ^19^ Ellagitannin (ET) and ellagic acid (EA)-rich diets such as pomegranate products are widely used as nutraceuticals. However, EA or ET have poor bioavailability and poor intestinal absorption *in vivo*. Recent studies highlighted that the overall benefits of pomegranate dietary supplements (PDS) were due to downstream metabolites generated from gut microbiota called “urolithins”.^20-25^ Urolithin A (UroA) is more bioavailable than its precursors and exhibits potent anti-inflammatory and gut barrier–protective effects.^23^ ^,^ ^26-29^ However, only 40–50% of individuals harbor the microbial community needed to produce UroA, limiting its natural generation. We and others have shown that UroA enhances gut barrier integrity, suppresses inflammatory responses, protects against colitis, and attenuates neutrophil-driven tissue damage.^30-32^ Our recent *in vitro* studies further demonstrated that UroA protects against iAs-induced cytotoxicity, apoptosis, oxidative stress, and epithelial barrier dysfunction in human intestinal organoids.^9^ These findings suggest that iAs-induced microbial dysbiosis may reduce beneficial microbial metabolites such as UroA, which are essential for maintaining gut barrier function and protecting against environmental toxins.

In mammals, iAs is primarily detoxified through enzymatic methylation mediated by arsenic (+3 oxidation state) methyltransferase (AS3MT). This enzyme uses S-adenosylmethionine (SAM) as a methyl donor to sequentially convert reduced arsenic (As^III^) into methylated metabolites, mainly monomethylarsonic acid (MMA) and dimethylarsinic acid (DMA).^33^ These reactions proceed through alternating reduction and oxidative methylation steps and result in metabolites that are more water-soluble and more efficiently excreted in urine, making methylation a central process in arsenic biotransformation. Although once viewed solely as a detoxification mechanism, arsenic methylation has important toxicological implications because some intermediate trivalent species (MMA^III^ and DMA^III^) are highly reactive and more toxic than inorganic arsenic.^34^ The less toxic pentavalent metabolites, methylarsonic acid (MAs^V^) and dimethylarsinic acid (DMAs^V^), are generated through spontaneous oxidation of their trivalent precursors and are efficiently eliminated from the body, primarily via urinary excretion.^35^ The balance and efficiency of AS3MT activity therefore strongly influences arsenic toxicity and disease risk. Genetic polymorphisms, nutritional factors affecting one-carbon metabolism (such as folate, vitamin B12, and choline), and epigenetic regulation all contribute to interindividual variability in arsenic metabolism and susceptibility to arsenic-related health effects.^36^

Humans and mice differ substantially in arsenic methylation efficiency. Mice methylate arsenic far more efficiently and therefore do not typically develop arsenic-related diseases seen in chronically exposed humans.^37^ ^,^ ^38^ To overcome this limitation, a humanized AS3MT mouse model (hAS3MT), in which the murine *As3mt* gene is replaced with the human *AS3MT* gene, was developed.^39^ These hAS3MT mice exhibit arsenic metabolism patterns where urinary MMA, DMA, and As tissue accumulations closely mimic human exposure profiles, making them a highly relevant *in vivo* model for studying arsenic toxicity.^40^ ^,^ ^41^ Using this humanized mouse model, we investigated the *in vivo* effects of iAs and gut protective activities of UroA on gut barrier function and inflammation to better understand UroA's therapeutic potential in mitigating arsenic-induced intestinal injury.

## Materials and methods

### Materials

General laboratory reagents were obtained from VWR (Radnor, PA), Thermo Fisher Scientific (Waltham, MA), Sigma-Aldrich (St. Louis, MO), and Fisher Scientific (Hampton, NH), unless otherwise specified. Urolithin A (UroA) was custom synthesized as previously described.^42^

### humanized *AS3MT* mice

The congenic B6N.129S6-Is(19BORCS7-AS3MT)1Bhk/Mmnc (RRID: MMRRC_069604-UNC) mice originated from the colony developed by Dr. Pardo-Manuel de Villena's laboratory at the University of North Carolina at Chapel Hill, NC, USA (UNC). Breeding pairs of these mice were provided for this study by Dr. Miroslav Stýblo (UNC). Mice were housed under specific pathogen-free (SPF) conditions with 12-hour light/dark cycles and provided food and water *ad libitum*. All procedures were approved by the Institutional Animal Care and Use Committee (IACUC), University of Louisville, KY, USA.

### iAs exposure and UroA treatment

Eight-week-old h*AS3MT* mice (both male and female) were acclimatized on a purified diet (Envigo Teklad; Cat. no. AIN-93G) for 3 weeks. Subsequently, mice were exposed to sodium arsenite (NaAsO_2_, 99% purity; Sigma-Aldrich) in drinking water (400  μg As/L) for 10 weeks. Control mice received arsenic-free deionized water. During exposure, mice were orally gavaged thrice weekly with either vehicle (1% carboxymethyl cellulose, 0.1% Tween-80) or UroA (20 mg/kg). The dosing regimen of UroA (20 mg/kg, orally, three times weekly) was selected based on prior studies demonstrating its efficacy in models of gut barrier dysfunction, including TNBS- and DSS-induced colitis.^30^ ^,^ ^42^ At this dose, UroA has been reported to improve intestinal barrier integrity and attenuate inflammation without evidence of toxicity. Thus, 20 mg/kg was chosen as a biologically effective and well-tolerated dose for *in vivo* administration. Spot urine (∼50–100 μL) and stool samples were collected weekly and at baseline and endpoint. Body weights were recorded weekly. At study termination, blood was collected for serum preparation, and spleen and mesenteric lymph nodes (mLNs) were harvested for immune analysis. Colon and small intestine were excised, photographed, and measured for length and weight. Tissues were either snap-frozen in liquid nitrogen for molecular analysis or fixed in 10% phosphate-buffered formalin for histology. In another cohort, hAS3MT mice (both male and female—6–8 weeks age) received either normal water or 100 ppb iAs in drinking water ad libitum for 28 weeks before sacrifice. They were fed an AIN-93G diet.

### Sample preparation and extraction of Short-chain fatty acids (SCFAs) from cecal content

Short-chain fatty acids (SCFAs) were extracted from lyophilized cecal content using an acidified aqueous extraction followed by solid–liquid extraction. Briefly, lyophilized cecal samples were pre-weighed and transferred into 2 mL Precellys bead tubes. They were extracted with 30 mM hydrochloric acid (HCl) containing 0.1 mM butyric acid-d₇ as the internal standard, using a sample-to-solvent ratio of 1:20 (w/v). The samples were homogenized using an Omniruptor for 2 min at maximum speed (30 Hz), ensuring efficient disruption of the matrix and release of SCFAs. Following homogenization, samples were incubated on ice for ~10 min to cool the extracts (minimizing enzymatic activity and metabolite instability). The homogenates were subsequently centrifuged at 15,000 rpm for 10 min at 4 °C, and the resulting supernatants were carefully transferred to clean Eppendorf tubes. The collected aqueous extracts were subjected to liquid–liquid extraction with an equal volume of chilled ethyl acetate (EtOAc) at a 1:1 (v/v) ratio to extract SCFAs. The mixtures were vortexed thoroughly for ~30 sec, incubated on ice for 5  min, and then centrifuged at 15,000 rpm for 1 min at 4 °C. This facilitated phase separation and enabled the partitioning of SCFAs into the organic phase. After centrifugation, the upper organic (EtOAc) layer was carefully collected and transferred to labeled clean Eppendorf tubes and stored at 4 °C until analysis. Finally, an aliquot of 70 µL of the EtOAc extract was transferred into a labeled autosampler vial with an insert, capped immediately, and shipped on dry ice to the IMAC facility at Texas A&M University for GC–MS/MS analysis.

### GC-MS/MS analysis of SCFAs

GC–MS/MS analysis of the EtOAc extracts or aliquots was performed using a triple quadrupole gas chromatography mass spectrometry system (TSQ EVO 8000, Thermo Scientific, Waltham, MA) for the detection and quantification of SCFAs. Chromatographic separation was achieved on a ZB WAX Plus, 30 m × 0.25 mm × 0.25 µm column (Phenomenex). Samples were maintained at room temperature on an autosampler before injection, and 1 µL of the extracted sample (EtOAc extract) was injected. The ionization was carried out in the electron impact (EI) mode at 70 eV. Sample acquisition and analysis were performed with TraceFinder 3.3 (Thermo Scientific). Final concentration of SCFAs was expressed as a function of the amount of cecal content.

### Sample preparation and extraction of Urolithin A from colon tissue

Preserved colon tissues from various cohorts were homogenized in fresh PBS at a ratio of 1:10 (w/v) (~50 mg tissue in 500 µL PBS). A 200 µL aliquot from each tissue homogenate sample was transferred to an Eppendorf tube and spiked with 10 µL of deuterated internal standard (Urolithin A-d_3_) solution from a 5 µg mL^−1^ stock. Protein precipitation and extraction of Urolithin A were performed twice using a 4× volume (~800 µL) of ice-cold methanol containing 0.1% formic acid, with vortex-mixing for 1–2 min after each extraction. Following centrifugation, the supernatants were collected, combined, and evaporated to dryness using SpeedVac. The dried extracts were reconstituted with 200 µL of ice-cold methanol:water (60:40, v/v) containing 0.01% formic acid, followed by a second centrifugation to remove any residual particulates. The resulting clarified supernatants were then transferred to an insert containing HPLC vials and subjected to high-resolution LC-MS analysis on an Orbitrap Exploris 120 mass spectrometry platform. Urolithin A was quantified against an instrumental calibration curve prepared in the reconstitution solvent (methanol/water, 60:40, v/v, containing 0.01% formic acid) with 250 ng mL^−1^ Urolithin A-d_3_ as the internal standard.

### High-resolution LC-MS/MS analysis and quantification of UroA

The high-resolution LC-MS/MS analysis was performed using a Thermo Scientific Vanquish UHPLC coupled to a Thermo Scientific Orbitrap Exploris 120 mass spectrometry (Thermo Scientific, FL, USA) platform at the University of Louisville. The system was controlled using Thermo Scientific Xcalibur software. The Vanquish UHPLC system is equipped with a binary pump and a thermostated autosampler set at 4 °C. Chromatographic separation was performed with a reverse-phase chromatographic column Phenomenex Kinetex 1.7 µm PFP 100 Å (100 × 2.1 mm, 1.7 µm particle size) at 40 °C. The column pressure was set at 800 bars. Mobile phase A consisted of LC-MS grade water containing 0.01% formic acid, while the mobile phase B consisted of LC-MS grade acetonitrile containing 0.01% formic acid. The injection volume was 3 μL. The mobile phases were delivered at a constant flow rate of 0.300 mL min^−1^ under binary gradient conditions as follows: 5% B (0.0–1.0 min), 15% B (3.5 min), 22% (5.5–6.3 min), 24% (6.95 min), 28% (8.7 min), 40% B (10 min), 55% (11 min), 95% B (12–13 min), followed by re-equilibration at 5% B from 13.5 to 15 min. The total run time was 15.0 min. Mass spectrometric detection of Urolithin A and its deuterated internal standards (Urolithin A-d_3_) was performed on an Orbitrap Exploris 120 mass spectrometer equipped with a heated electrospray ionization (H-ESI) source and operated in negative ionization mode. Source parameters were set as follows: spray voltage 2500 V, sheath gas 50 (Arb), auxiliary gas 10 (Arb), sweep gas 1 (Arb), ion transfer tube temperature 325 °C, and vaporizer temperature 350 °C. The expected LC peak width was set to 6 sec. Urolithin A and the internal standard Urolithin A-d_3_ were monitored by targeted selected ion monitoring (tSIM) at an Orbitrap resolution of 60,000 using precursor ions with central masses of m/z 227.035 (z = 1) and m/z 230.054 (z = 1), respectively, over the 0–15 min acquisition window. The confirmation of Urolithin A and Urolithin A-d_3_ was achieved by targeted MS/MS (tMS^2^) using HCD with stepped normalized collision energies of 60%, 65%, and 70%. Product ion spectra were acquired over an m/z range of 100–450 at an Orbitrap resolution of 30,000. The isolation window was set to 0.5 m/z for both the tSIM and tMS^2^ acquisitions. Data were acquired in profile mode with the RF lens set to 70%, AGC target set to standard, maximum injection time set to auto, and microscans set to 1 for both tSIM and tMS^2^ acquisitions. Urolithin A was identified by accurate mass, retention time, and confirmatory MS/MS fragmentation matching to the reference standard. Quantification of Urolithin A was performed in TraceFinder 5.2 (Thermo Scientific) using a tMS^2^-based workflow with deuterated labeled internal standard normalization. The analytical response was expressed as the peak area ratio of analyte to internal standard and converted to extracted amounts using an instrumental calibration curve prepared over a concentration range of 1–1000 ng mL^−1^. Calibration standards were prepared in (methanol:water, 60:40, v/v) containing 0.01%formic acid and the internal standard Urolithin A-d_3_ at a final concentration of 250 ng mL^−1^. The extracted amounts were subsequently normalized to tissue weight and expressed accordingly in the result.

### Histopathology

Colon tissues were fixed in 4% formalin for 16 hours, transferred to 70% ethanol, embedded in paraffin, and sectioned at 5 μm as described previously.^31^ Sections were stained with hematoxylin and eosin (H&E) by Saffron Scientific Histology Services (IL, USA). Images were captured using a PanDesk Slide Scanner (3DHISTECH Ltd., MI, USA), and inflammation was scored using a standard index.^43^

### Intestinal permeability assay

Gut barrier integrity was assessed using the FITC–dextran assay. Mice were fasted overnight prior to oral gavage with FITC–dextran (4 kDa; Sigma-Aldrich) at 60 mg per 100 g body weight, 4 hours before euthanasia. Serum FITC–dextran concentrations were quantified against a standard curve (FITC-dextran standards were prepared in mouse serum) using a SpectraMax iD3 microplate reader (Molecular Devices, CA) at 485/525 nm.

### Western blot analysis

Colon tissues were homogenized in RIPA buffer containing protease inhibitors (Sigma-Aldrich). Lysates were processed for immunoblotting as previously described.^44^ Protein bands were visualized using chemiluminescent substrate and imaged on a Bio-Rad ChemiDoc Imaging System (Hercules, CA). Antibody details are provided in Supplementary Table 1.

### Real-time PCR

Total RNA was extracted from colon tissues, and relative mRNA expression of tight junction proteins and cytokines was quantified using SYBR Green-based qPCR as previously described.^45^ Data were normalized to *β*-actin and analyzed using the ^-ΔΔ^CT method. Primers were purchased from realtimeprimers.com.

### Immunofluorescence

Paraffin-embedded colon sections were stained with primary antibodies against MUC2 (1:250; GeneTex, Cat# GTX100664) and ZO-1 (1:100; Invitrogen, Cat# 33-9100), followed by Alexa Fluor-conjugated secondary antibodies (Invitrogen) as mentioned elsewhere.^32^ Sections were mounted with VECTASHIELD antifade medium containing DAPI and imaged using an Olympus VS200 Slide Scanner. Quantification was performed using ImageJ.

### Oxidative stress marker (4-HNE) staining

To detect the ROS levels by 4HNE immunohistochemistry colon sections were stained with 4HNE antibody (1:500 dilution, Alpha Diagnostic International, Cat# HNE11-S). Staining protocol was carried out as described previously.^46^ The 4HNE -stained tissue sections were imaged using PanDesk Slide Scanner (3DHISTECH Ltd., MI, USA).

### Myeloperoxidase (MPO) activity

MPO activity was measured in colon homogenates using an MPO Assay Kit (Abcam) per manufacturer's instructions. Absorbance was recorded at 412 nm using a SpectraMax iD3 reader.

### Cytokine analysis (ELISA)

Levels of IL-6, TNF-*α*, and IL-1β in serum and colon homogenates were quantified using ELISA kits (BioLegend) according to manufacturer's protocols. Absorbance was measured at 450 nm and 570 nm.

### Flow cytometry

Single-cell suspensions from spleen and mLNs were prepared by mechanical dissociation and filtration through 70 μm strainers. After Fc blocking, cells were stained with fluorochrome-conjugated antibodies against CD45, CD11b, and Ly6G. Data were acquired on a BD FACSCanto II and analyzed using FlowJo software. Antibody details are listed in Supplementary Table 2.

### Microbiota sequencing and analysis

Fecal DNA was extracted using the QIAamp PowerFecal Pro DNA Kit (Qiagen). The V3–V4 region of the 16S rRNA gene was amplified using barcoded primers and sequenced on the Illumina platform at Novogene Corporation Inc. Raw sequencing data were processed using QIIME (www.qiime.org). Briefly, reads were quality filtered to remove low-quality sequences, followed by dereplication and inference of amplicon sequence variants (ASVs) using DADA2^47^ (Li et al., 2020). ASVs were taxonomically classified against the Greengenes database. Beta diversity was assessed using UniFrac distance metrics, and statistical significance was determined by PERMANOVA. Statistical modeling of individual genera was performed using linear models and the microViz package.^48^

### Determination of arsenic levels

All digestion tubes (Eppendorf or metal-free tubes) were pretreated by soaking in 10% nitric acid overnight, followed by rinsing five times with deionized (DI) water and air-drying. Urine samples (100 µL) were transferred into the prepared digestion tubes, and 300 µL of 70% trace-metal–grade nitric acid (Fisher Scientific, Cat# A509P500) was added. Samples were digested in a 65 °C incubation shaker for 4 h until the solutions became clear. The digested solutions were then carefully transferred into 4 mL of DI water to achieve a final nitric acid concentration below 5%, mixed thoroughly, and filtered through a 45 µm cell strainer into standard 15 mL metal-free tubes (VWR, Cat# 89049-172). For assay blanks and calibration standards, six 35 mL tubes containing 5% nitric acid were prepared by mixing 32.5 mL DI water with 2.5 mL of 70% nitric acid (VWR, Cat# 89049-176). Samples were analyzed using an Agilent 7800 ICP-MS (Agilent Technologies, Japan), with instrument performance optimized using a 1 ppb tuning solution and auto-tuned with a 10 ppb tuning solution (Agilent, Cat# 5188-6564). Samples were introduced via an SPS 4 autosampler, and calibration standards were obtained from Inorganic Ventures (Cat# IV-STOCK-50). Data acquisition was performed using Agilent MassHunter software in helium collision mode, with each sample measured in triplicate and mean values used for analysis.

### Statistical analysis

Data were analyzed using GraphPad Prism 10 (GraphPad Software, San Diego, USA). Comparisons were performed using unpaired t-tests or one-way ANOVA followed by Tukey's multiple comparisons test. Results are presented as mean ± SEM. Significance was defined as ****p < 0.0001; ***p < 0.001; **p < 0.01; *p < 0.05; ns = not significant.

## Results

### UroA protects against iAs-induced intestinal injury in h*AS3MT* mice

**Figure 1.**
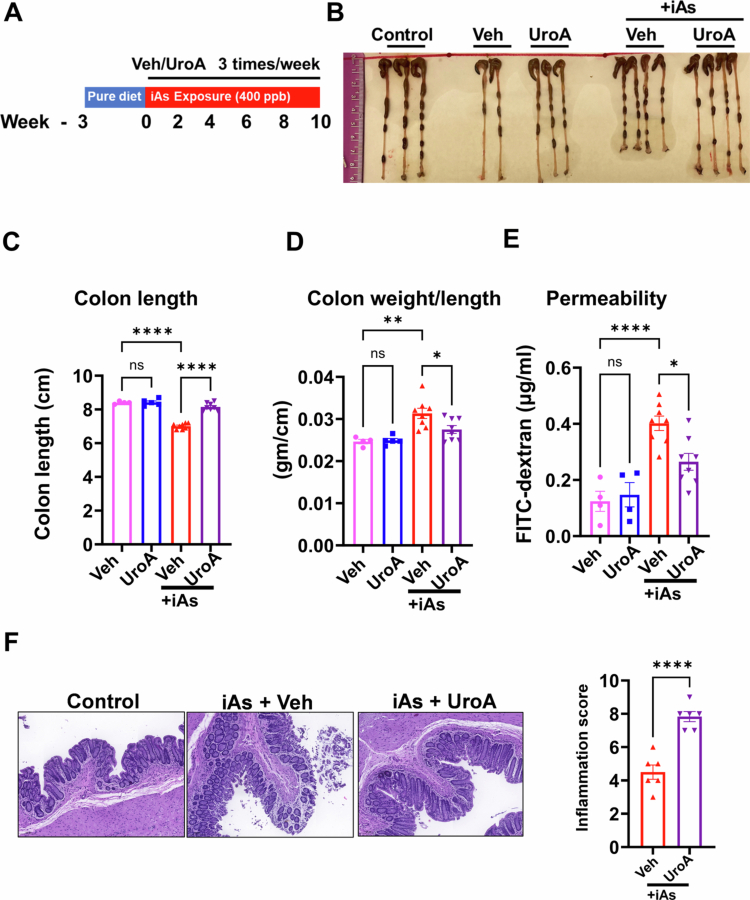
UroA protects against iAs-induced gut injury and gut permeability. A. Experimental design illustrating iAs^III^ exposure and UroA-treatment regimen. The experimental design shows that the mice were maintained on a purified diet from week −3 to week 0. Beginning at week 0 and continuing through week 10, the mice received either vehicle (Veh) or Urolithin A (UroA) three times per week, along with or without exposure to inorganic arsenic (iAs) at 400 ppbB. Gross images of colons of h*AS3MT* mice exposed to iAS and treated with or Vehicile UroA. Colons of control h*AS3MT* mice (not on purified AIN-93G diet) as well as colons of h*AS3MT* mice that were not exposed to iAS (Veh, UroA) are shown. C. Colon lengths D. Ratios of colon weight/length (gm/cm)E. Gut permeability determined by FITC-Dextran assay as decribed in methods. F. Hematoxylin and eosin (H&E) stained sections of colons are shown. Inflammation scores are shown. Scale bar indicates 100 μm. Statistics performed using one-way ANOVA in GraphPad Prism software. *p < 0.05, **p < 0.01, ****p < 0.0001. Error bar, mean ± SEM.

The h*AS3MT* mouse model replicates human arsenic metabolism, providing a relevant system for investigating the in vivo effects of iAs.^40^ ^,^ ^41^ To assess the impact of iAs on gut barrier function, h*AS3MT* mice were exposed to environmentally relevant concentrations of iAs (100 ppb; 100 μg/L) in drinking water for 28 weeks. As shown in Supplementary Figure 1, iAs exposure markedly reduced the expression of tight junction proteins, including zonula occludin (ZO-1), occludin (OCLN), and claudin-4 (CLDN-4) leading to compromised intestinal barrier integrity. We next evaluated whether UroA mitigates iAs-induced barrier dysfunction. Mice were maintained on a purified diet for three weeks to limit basal arsenic contamination, then exposed to iAs (Na₃AsO₄, 400 ppb) for 10 weeks, a dose reflecting chronic human exposure in certain geographical regions such as Chile.^49^ During exposure, mice received vehicle (1% carboxymethyl cellulose, 0.1% Tween-80) or UroA (20 mg/kg) by oral gavage three times weekly (Figure 1A). UroA levels in mouse colonic tissue were quantified using LC–MS, as described in the Methods. Representative chromatograms of UroA peaks alongside analytical standards are shown in Supplementary Figure 2A. As expected, UroA was detected exclusively in mice administered UroA, whereas no UroA was detectable in vehicle- or iAs-treated mice (Supplementary Figure 2B). These findings further indicate the absence of endogenous UroA production in the colons of these mice.

Body weight remained unchanged across groups (Supplementary Figure 3A). Urinary arsenic levels increased following iAs exposure, with no significant differences between vehicle and UroA-treated mice (Supplementary Figure 3B). Shortening of colon length and increased colon weights represent increased inflammation in colon tissues. As shown in Figure 1B−D, iAs exposure (400 ppb; 10 weeks) resulted in shortened colons and increased colon weight-to-length ratios compared to control group. UroA treatment preserved colon length and reduced weight-to-length ratios, suggesting attenuation of iAs-induced inflammation (Figure 1B−D). Gut permeability, measured by an *in vivo* FITC–dextran assay, was significantly increased in iAs‑exposed mice; however, this iAs‑induced increase in FITC–dextran permeability was markedly attenuated by UroA treatment (Figure 1E). Histological analysis revealed epithelial cell damage and inflammatory cell infiltration in iAs-exposed colons, whereas UroA-treated mice exhibited improved tissue architecture and reduced inflammation (Figure 1F). Collectively, these findings demonstrate that UroA confers protection against iAs-induced gut barrier dysfunction and inflammation *in vivo*.

### UroA reverses the reduction of tight junction proteins and mucin caused by iAs exposure

**Figure 2.**
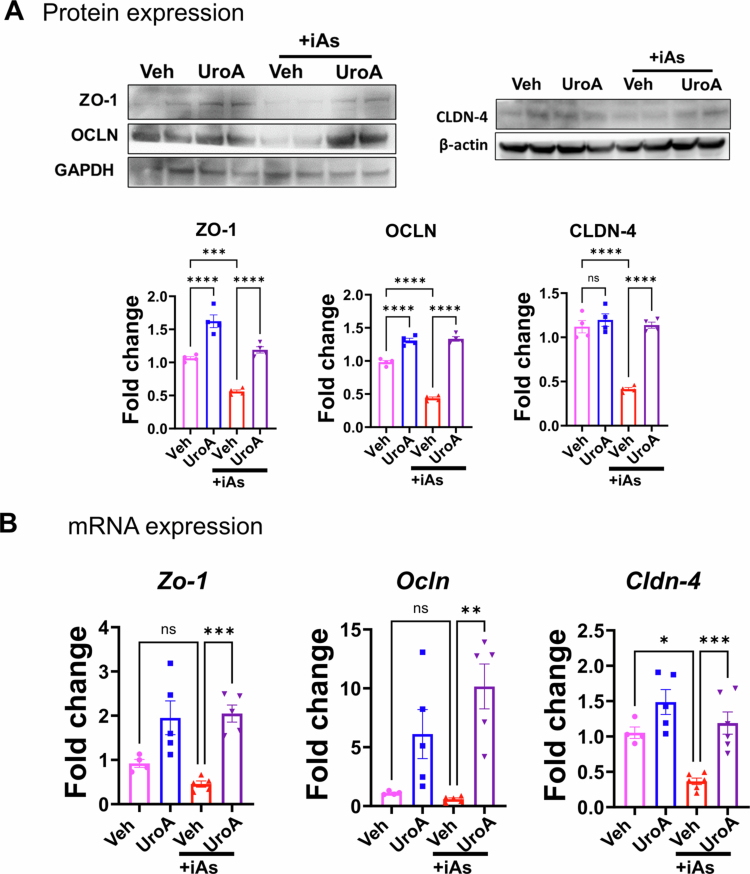
UroA attenuated iAs-induced downregulation of tight junctional proteins at protein and mRNA levels A. Representative Westen blots showing the expression of Zonula occluden 1 (ZO-1), occludin (OCLN) and claudin 4 (CLDN-4) proteins in the colons of h*AS3MT* mice. The Western blots (*n* = 4) were quantified by using Image J software. B. The fold changes of mRNA levels of Z*o1*, *Ocln*, and *Cldn4* genes in the colons were determined by SyBR green RT-PCR method. Statistics were performed using one-way ANOVA in GraphPad Prism software. **p < 0.01, ***p < 0.001, ****p < 0.0001. Error bar, mean ± SEM.

**Figure 3.**
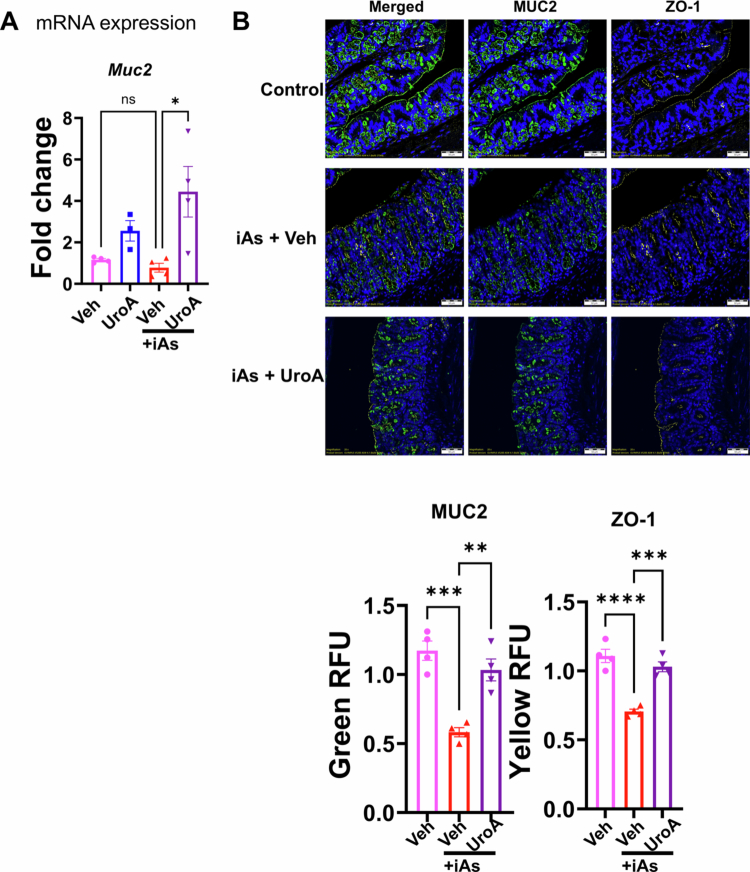
UroA reduced iAs-induced downregulation of mucin and tight junctional proteins. A. The fold changes in mRNA levels of *Muc2* in the colons were determined by SyBR green RT-PCR method. B. Colon sections were stained with anti-MUC2 and anti- ZO-1 antibody followed by secondary antibody tagged with Alexa Fluor 488 (green). and Alexa Fluor 594 (yellow), respectively. The nucleus was stained with DAPI (blue). The fluorescence images were captured using VS200 slide scanner. The scale bar indicates 50 μm. Red asterisk represents a damaged barrier that did not stain for ZO-1. Fluorescence intensity was quantified using Image J. Statistics were performed using one-way ANOVA in GraphPad Prism software. **p < 0.01, ***p < 0.001, ****p < 0.0001. Error bar, mean ± SEM.

To investigate the potential mechanisms underlying UroA-mediated attenuation of iAs-induced intestinal damage, we first evaluated the levels of junctional proteins. iAs significantly decreased tight junction proteins (TJPs) such as ZO-1, OCLN and CLDN-4 in the colon both at the protein and mRNA level, while UroA treatment protected from iAs-induced downregulation (Figure 2A and B). Next, to determine whether iAs affected the mucin in colon, we evaluated the expression of MUC2. The mRNA analysis revealed that exposure to iAs in mice downregulated *Muc2* expression and that UroA restored expression (Figure 3A). To confirm the expression patterns of ZO-1 and MUC2 in colons, we performed immunofluorescence staining. As shown in Figure 3B, UroA preserved ZO-1 localization and mucins, which were disrupted by iAs. Although MUC2 expression was altered, no significant changes in goblet cell numbers were observed across the experimental groups, indicating that UroA primarily modulates mucus production rather than goblet cell abundance (data not shown).

### UroA treatment protects against iAs-induced oxidative stress and inflammation in h*AS3MT* mice colons

**Figure 4.**
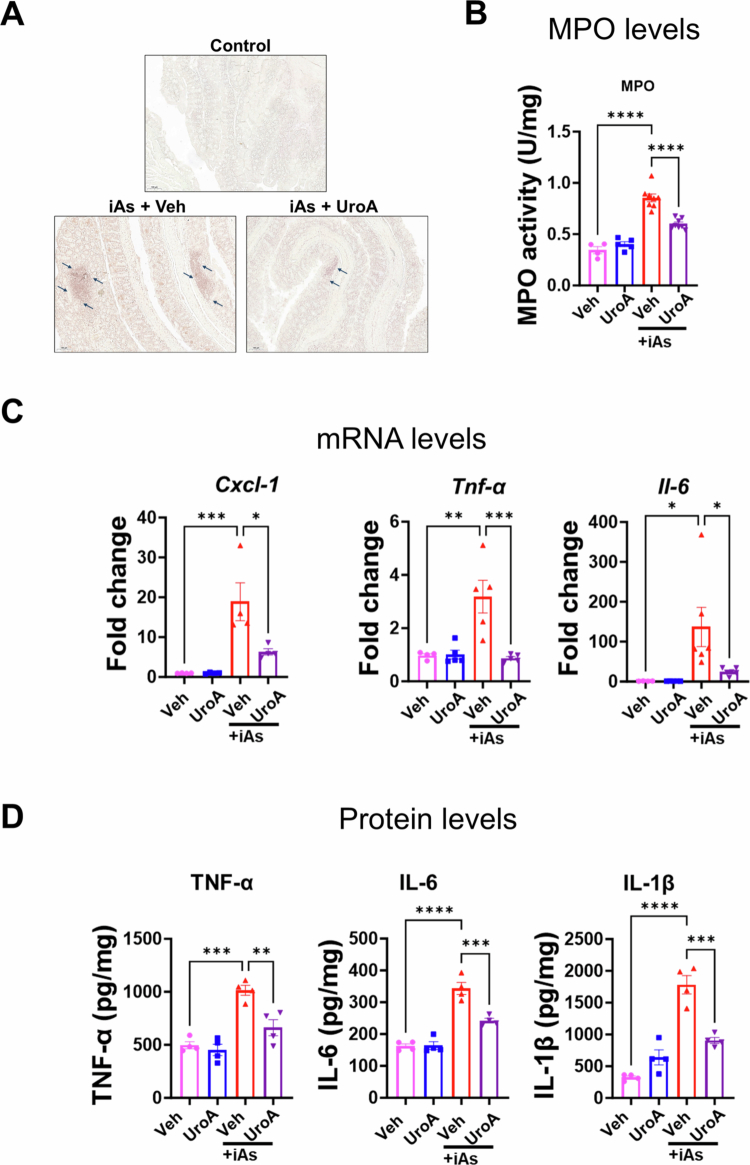
UroA treatment protected against iAs-induced ROS generation and inflammation in h*AS3MT* mice colon. A. Detection of elevated ROS levels by 4HNE immunohistochemistry. Blue arrow shows oxidative stress-mediated lipid oxidation in colon sections. Scale bar 100 μM. B. MPO activity in colon tissues was measured using MPO activity assay kit (Abcam). C. The fold changes in mRNA levels of neutrophil-recruiting chemokine (*Cxcl-1*) inflammatory cytokine genes (*Tnf-α* and *Il-6*) in the colons were determined by the SYBR Green RT-PCR method. D. Inflammatory cytokines from colon tissues were determined by ELISA kits. Statistics were performed using one-way ANOVA in GraphPad Prism software. **p < 0.01, ***p < 0.001, ****p < 0.0001. Error bar, mean ± SEM.

Exposure to iAs is known to increase oxidative stress and inflammation, particularly in individuals with inflammatory bowel disease.^50^ Our previous study demonstrated that UroA significantly reduces iAs-induced oxidative stress *in vitro.* ^9^ To assess this effect *in vivo*, we examined the formation of 4-hydroxynonenal (4-HNE), a marker of lipid peroxidation, and an indicator of reactive oxygen species (ROS), in the colons of experimental mice. Immunohistochemistry revealed elevated 4-HNE levels in iAs-exposed mice, indicating localized tissue damage caused by arsenic-induced oxidative stress (Figure 4A). UroA treatment markedly reduced 4-HNE accumulation, protecting the colon from ROS-mediated injury (Figure 4A). Next, we evaluated myeloperoxidase (MPO) activity, a neutrophil marker, and CXCL1, which regulates neutrophil recruitment. iAs exposure significantly increased MPO activity and *Cxcl-1* gene expression, implicating increased neutrophil activity in the colons. In contrast, UroA treatment attenuated neutrophil activity and recruitment, thereby reducing colonic inflammation (Figure 4B−C). Given the role of iAs in promoting inflammation, we further investigated whether UroA modulates inflammatory cytokine responses during iAs exposure in colons of these mice. iAs exposure led to a marked increase in pro-inflammatory cytokines (IL-6, TNF-*α*, IL-1β) in colon homogenates (Figure 4C−D). UroA treatment significantly decreased these cytokines at both mRNA and protein levels (Figure 4C−D). Collectively, these findings indicate that UroA mitigates iAs-induced oxidative stress, neutrophil activation, and inflammatory cytokine production, thereby protecting colonic tissue integrity.

### UroA administration attenuates iAs-induced systemic inflammation in h*AS3MT* mice

**Figure 5.**
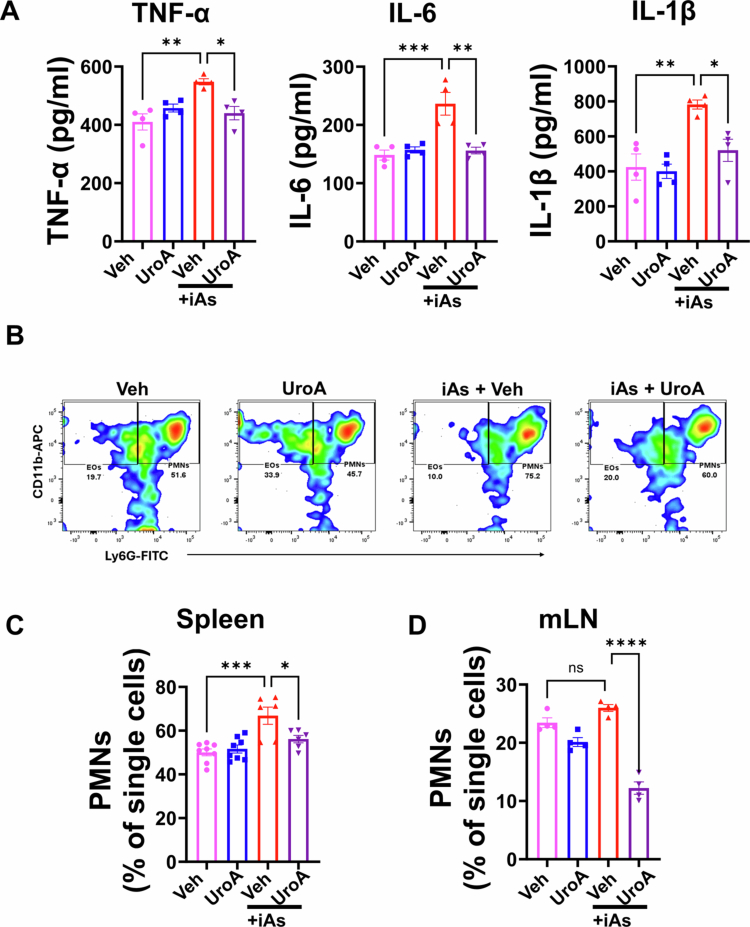
UroA administration inhibited iAs-induced systemic inflammation in *hAS3MT* mice. A. Inflammatory cytokines from serum were determined by ELISA kits. B. Representative flowcytometry scatter plot indicating iAs exposure led increase in neutrophils. Gating strategy is provided in Supplementary Figure 4. C. Immune cells from spleen and mesenteric lymph nodes of experimental mice were analyzed using standard flow cytometric procedures. The percentages of PMNs are shown. Statistics were performed using one-way ANOVA in GraphPad Prism software. **p < 0.01, ***p < 0.001, ****p < 0.0001. Er radiation-induced injury ror bar, mean ± SEM.

To determine whether iAs exposure triggers systemic inflammation, we measured serum levels of key pro-inflammatory cytokines (IL-6, TNF-*α*, and IL-1β). As expected, iAs exposure significantly elevated these cytokines, whereas UroA treatment markedly reduced their levels, indicating effective mitigation of systemic inflammation (Figure 5A). Since iAs increased levels of MPO (a marker for neutrophil activities) in colons, we next examined whether iAs increased systemic polymorphonuclear leukocytes (PMNs) using flow cytometry methods. We have quantified PMN populations in spleen and mesenteric lymph nodes by flow cytometry. iAs exposure significantly increased PMN cell infiltration in the spleen, whereas UroA treatment significantly reduced PMN numbers in both the spleen and MLNs of iAs-exposed mice, indicating attenuation of the systemic inflammatory burden (Figure 5B−D).

### UroA restores the iAs-induced microbial dysbiosis

**Figure 6.**
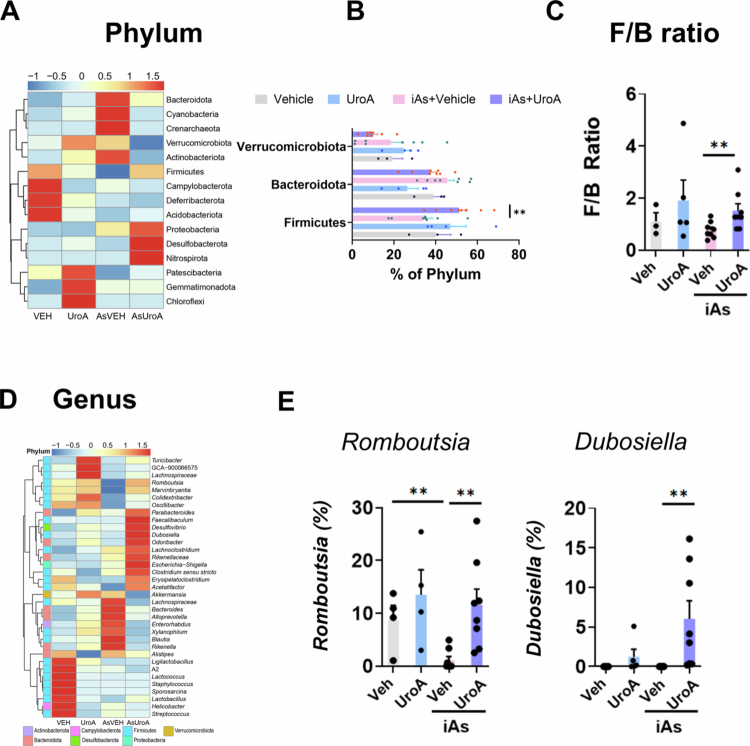
UroA treatment corrects iAs-induced gut microbial dysbiosis. h*AS3MT* mice were exposed to iAs as described in Figure 2. Stool samples were collected at the end of the experiment and isolated bacterial gDNA. The 16S rRNA V3-V4 regions were sequenced using Ilumina platform at Novogene. The bacterial analysis is carried out as described in methods. A. Heat map at phylum levels of vehicle, iAs, UroA and iAs + UroA groups. B. Bar graph of representing phylum levels. C. Ratio of Firmicutes and Bacteroides (F/B) D. Heat map representing comparisons between groups at genus levels. E. Levels of *Romboutsia* and *Dubosiella* genus are shown.

To determine whether iAs^3^⁺ exposure alters gut microbiota composition, we performed 16S rRNA gene sequencing (V3–V4 regions) of stool samples from h*AS3MT* mice exposed to iAs with or without UroA treatment. iAs exposure induced significant shifts in microbial communities, characterized by a reduction in the Firmicutes phylum and a decreased Firmicutes/Bacteroidetes (F/B) ratio. As shown in Figure 6A−C, upon UroA treatment improved Firmicutes abundance and F/B ratio in iAs-exposed mice. A lower F/B ratio is a metric frequently associated with gastrointestinal disorders such as IBD.^51^ Improvement of the F/B ratio by UroA suggests potential benefits for gut health. At the genus level, iAs exposure markedly reduced beneficial taxa including *Romboutsia, Dubosiella*, *Faecalibaculum*, and *Odoribacter* (Figure 6D), Supplementary Table 3). These genera are known contributors to short-chain fatty acid (SCFA) production and regulators of inflammatory pathways.^52^ Importantly, UroA treatment protected against iAs-induced depletion of SCFA-producing bacteria (Supplementary Table 4). Notably, most iAs-induced alterations were reversed following UroA administration, as indicated by restoration of reduced taxa and normalization of previously upregulated taxa. For example, UroA increased the levels of *Romboutsia* and *Dubosiella* which are known to provide beneficial metabolites to maintain gut barrier integrity (Figure 6E). Further, functional analysis of these beneficial activities is yet to be established in iAs-induced gut barrier dysfunction.

**Figure 7.**
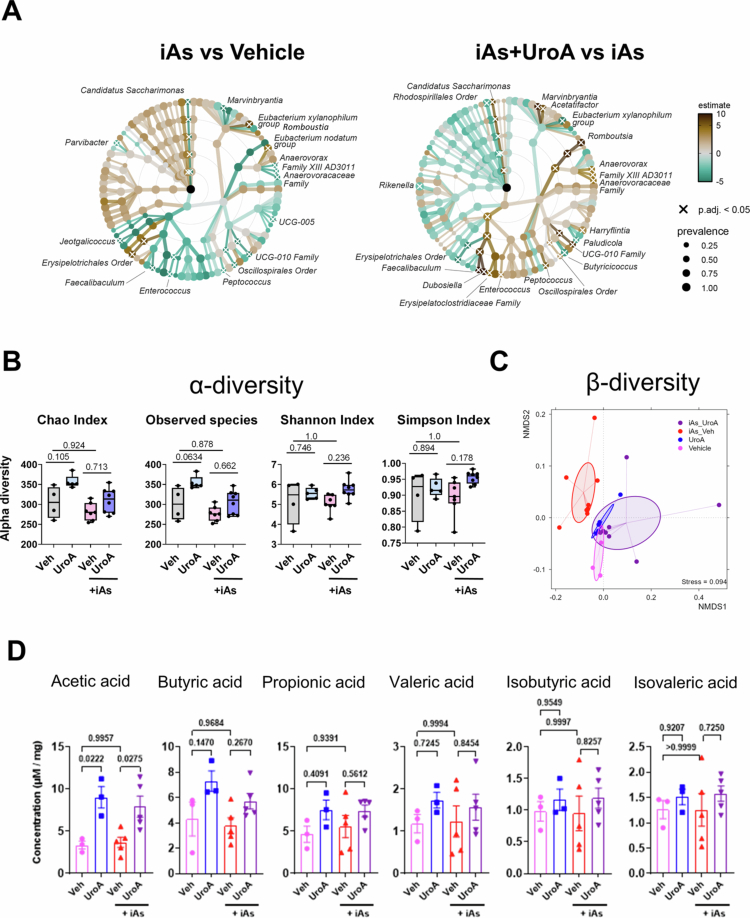
Taxonomic association trees showing differential abundance patterns under arsenic exposure and UroA treatment. A. Left panel illustrates associations for arsenic exposure (Control-Veh vs. Arsenic-Veh), while right panel shows associations for UroA treatment (Arsenic-Veh vs. Arsenic-UroA). Each node represents a taxon aggregated at the indicated rank from phylum (innermost ring) to genera (outer-most ring). Node color encodes the estimated effect size (linear model coefficient) for the specified contrast, with brown tones indicating positive associations and blue tones indicating negative associations. Node size reflects taxon prevalence across all samples. Edges connect taxa to their parent nodes in the taxonomic hierarchy. Models were fit using the microViz R package with log₂ abundance transformation after zero replacement. Only taxa with an FDR-adjusted *p* < 0.05 are displayed. B. Alpha diversity among indicated treatments is analyzed as described in methods. C. Beta diversity plot: Principal coordinate analysis plot (PCoA) using unweighted UniFrac distance of bacterial composition in mice exposed to with or without iAs and treatments with vehicle or UroA. D. Short chain fatty acids are quantified from cecal content of indicated groups using GC-MS/MS methods.

Differential abundance analysis using taxon-wise linear models of log2-transformed relative abundances confirmed widespread iAs-induced shifts across multiple bacterial clades and demonstrated partial restoration of several depleted taxa in UroA-treated animals (Figure 7A; Supplementary Table 5). To assess whether UroA mitigated iAs-associated reductions in alpha diversity, richness and diversity metrics were compared between UroA- and vehicle-treated animals within the iAs-exposed group (Figure 7B). Although UroA-treated animals exhibited modestly higher richness and diversity, these differences did not reach statistical significance. Consistent with these findings, two-way ANOVA revealed no significant iAs × treatment interaction across alpha diversity measures, indicating additive rather than interactive effects of arsenic exposure and UroA treatment. Beta-diversity analysis using unweighted UniFrac distances demonstrated clear separation among vehicle, iAs, and iAs + UroA groups (Figure 7C). Permutational multivariate analysis of variance (PERMANOVA) confirmed that iAs exposure significantly altered overall community composition, while UroA treatment partially reversed iAs-associated dysbiosis. Together, these results suggest that while UroA does not fully restore community structure, it exerts a measurable corrective effect on arsenic-induced microbial restructuring at the compositional and taxonomic levels.

Next, we analyzed short chain fatty acid (SCFA) composition in cecal content using GC-MS/MS methods. As shown in Figure 7D, UroA treatment significantly increased acetic acid levels compared with vehicle treatment. Although butyrate levels showed an upward trend following UroA treatment, this difference did not reach statistical significance. No significant changes were observed in the other measured short‑chain fatty acids.

## Discussion

iAs is classified by the U.S. Environmental Protection Agency (EPA) as a top-priority hazardous substance, affecting over 230 million people worldwide and contributing to multiple health problems that often lack effective treatments.^53^ Chronic iAs exposure is associated with cancer and severe gastrointestinal (GI) disorders, including inflammatory bowel disease (IBD).^54^ Epidemiological studies suggest a potential association between iAs exposure and increased risk of IBD, particularly in regions with chronic groundwater contamination. Elevated arsenic levels in drinking water have been documented in areas such as Bangladesh and West Bengal (India), Taiwan, northern Chile, and parts of the southwestern United States, where higher burdens of gastrointestinal morbidity and chronic intestinal inflammation have been reported. Population-based studies from these regions have linked long-term iAs exposure to persistent gastrointestinal symptoms, immune dysregulation, and inflammatory conditions consistent with IBD pathophysiology. Notably, early-life and chronic exposures appear to confer heightened vulnerability, aligning with evidence that arsenic can induce sustained immune and epithelial alterations.^55^ ^,^ ^56^ Despite confounding factors, geographic overlap and experimental evidence suggest iAs is an underrecognized contributor to IBD susceptibility and severity.

The toxicity and environmental fate of iAs depend on its chemical speciation, which is influenced by microbial and host metabolism. Importantly, iAs toxicity differs between humans and laboratory mice due to variations in arsenic metabolism and gut microbiota, necessitating the use of human-relevant models for mechanistic studies. Conventional laboratory mice exhibit limited susceptibility to iAs-induced effects at doses relevant to human exposure because of their more efficient arsenic methylation. To overcome this limitation, we employed hAS3MT mice, which express the human arsenite methyltransferase enzyme, to evaluate the impact of chronic iAs exposure on gut barrier integrity and microbiota.^57^

The intestinal gut barrier plays a critical role in maintaining host homeostasis by regulating the selective passage of nutrients, metabolites, and microbial products while preventing the translocation of harmful luminal antigens and pathogens.^29^ ^,^ ^58^ This barrier is composed of a mucus layer, a tightly regulated epithelial cell layer connected by intercellular junctional complexes and underlying immune components that collectively preserve intestinal integrity. Disruption of gut barrier function can lead to increased intestinal permeability, commonly referred to as “leaky gut,” which has been implicated in the development of local and systemic inflammatory, metabolic, and immune-mediated diseases such as IBD.^29^ Consequently, maintaining gut barrier integrity is essential for overall health, and environmental factors that compromise this barrier may have significant pathophysiological consequences. Our previous studies using *in vitro* models demonstrated that exposure to iAs leads to compromise gut barrier function by altering tight junction integrity, increasing epithelial permeability, and inducing oxidative stress and inflammation.^54^ ^,^ ^59^ However, direct effects of iAs on gut barrier functions and its mitigation strategies *in vivo* are not well-established due to lack of appropriate mouse models. Here, we used an appropriate human-relevant mouse model, h*AS3MT* to investigate iAs-exposure on gut barrier activities.

Our findings suggest that chronic iAs exposure downregulates gut epithelial tight junction proteins (TJPs), increases intestinal permeability, and promotes inflammation *in vivo*. Further, treatment with microbial metabolite UroA protected from iAs-induced gut barrier dysfunction. Specifically, iAs exposure reduced expression of tight junction proteins, ZO-1, OCLN, and CLDN-4, accompanied by increased FITC–dextran leakage and histological evidence of epithelial damage, which are hallmarks of barrier dysfunction. Disruption of tight junction integrity is known to facilitate translocation of luminal antigens and microbial products, thereby exacerbating intestinal and systemic inflammatory signaling.^60^ Previously, we have shown that iAs-induced gut barrier dysfunction is driven by iAs-induced oxidative stress, mitochondrial dysfunction, and activation of proinflammatory signaling pathways in intestinal epithelial cells. The data presented here *in vivo* also suggests that iAs exposure alters TJPs expression, mucosal immune responses (increased levels of PMN) and oxidative stress that potentially cause increased barrier dysfunction.

UroA is a microbiota-derived metabolite produced from dietary ellagitannins (ETs) and ellagic acid (EA), and its generation requires both adequate intake of ET/EA-rich foods (e.g., pomegranate, berries, and walnuts) and the presence of specific gut microbial taxa. Our previous and current observations indicate that mice housed in our facility do not produce detectable levels of UroA, even following EA supplementation (unpublished data), suggesting the absence of UroA-producing microbes in this model. Consistent with this, UroA production is highly variable in humans, with only ~35%–50% of individuals capable of generating UroA after consumption of pomegranate juice, reflecting interindividual differences in gut microbiota composition. Collectively, these findings highlight that endogenous UroA production is diet- and microbiota-dependent and not universally conserved across species or individuals. Therefore, to directly assess the biological effects of UroA independent of microbial conversion capacity, we employed exogenous UroA supplementation in our mouse studies. Therefore, the effects observed in this study are microbiota-independent with respect to UroA production, while remaining relevant to understanding UroA-mediated mechanisms *in vivo.*

Importantly, UroA administration restored tight junction disruption at both protein and transcript levels, reduced inflammation, and the overall PMN population. Similar protective effects were observed for MUC2, a key component of the mucus layer, indicating improved luminal defense when treated with UroA. Preservation of the mucus barrier likely contributes to reduced epithelial injury and inflammatory activation, further supporting intestinal homeostasis.^61^ The combined improvement in tight junction integrity, mucus production, and reduced inflammation underscores the multifaceted role of UroA in gut barrier protection.

Oxidative stress is a major driver of iAs toxicity, often leading to lipid peroxidation, cytokine release, and chronic inflammation.^62^ Consistent with this, iAs exposure markedly increased 4-HNE accumulation, a marker of ROS-mediated damage, whereas UroA significantly reduced 4-HNE staining, confirming its antioxidant properties in vivo. Furthermore, iAs induced robust inflammatory responses, including elevated IL-6, TNF-*α*, IL-1β, CXCL1, and MPO activity, which contribute to mucosal injury and neutrophil recruitment. UroA attenuated these responses at both transcriptional and protein levels, highlighting its ability to modulate inflammatory cascades and protect against immune-driven tissue damage. Beyond local GI injury, chronic iAs exposure promoted systemic inflammation, as evidenced by increased serum cytokines and elevated PMN in spleen and lymph nodes. UroA significantly reduced circulating cytokines and restricted PMN infiltration, underscoring its immunomodulatory potential and capacity to mitigate systemic consequences of chronic toxic exposure. By targeting interconnected pathways of ROS generation and inflammation, UroA emerges as a promising microbiota-derived metabolite with therapeutic potential for protecting against arsenic-induced toxicity and its associated inflammatory sequelae. Further studies are warranted to elucidate the precise molecular mechanisms underlying UroA's antioxidant and immunoregulatory actions and to evaluate its translational relevance in human populations exposed to environmental arsenic.

A key observation from this study is that iAs disrupts gut microbiota composition, reducing Firmicutes abundance and the Firmicutes/Bacteroidetes (F/B) ratio, changes commonly linked to GI disorders such as IBD.^51^ UroA treatment partially restored Firmicutes abundance and normalized the Firmicutes-to-Bacteroidetes ratio, suggesting improved microbial homeostasis. iAs exposure reduced several beneficial taxa, including *Romboutsia*, *Dubosiella*, *Faecalibaculum*, and *Odoribacter*, which are commonly associated with short-chain fatty acid (SCFA) production and gut barrier support. (Supplementary Table 4). Consistent with previous reports, depletion of these genera likely contributes to impaired epithelial integrity and low-grade inflammation. The capacity to reduce arsenic in the gut has been linked to bacteria harboring the arsC gene, which encodes arsenate reductase.^63^ Although 16S rRNA gene sequencing does not enable direct inference of functional gene content, we assessed the relative abundance of bacterial genera previously associated with arsenic transformation, including *Aeromonas*, *Pseudomonas*, and *Corynebacterium*. No significant differences in the abundance of these taxa were detected across experimental groups. Notably, UroA treatment increased several of these beneficial microbes, which may enhance intestinal barrier function through increased production of protective microbial metabolites such as SCFAs and indole derivatives. Previously, it was shown that UroA treatment led to increase in beneficial metabolites such as SCFAs and indole-3-aldehyde.^64^ These metabolites are known to improve gut barrier functions. Further, SCFA analysis suggests that UroA treatment led to an increase in acetic acid and butyrate levels (increased trend but did not reach statistical significance), suggesting potential benefits. Acetate, a major microbiota-derived short-chain fatty acid, strengthens the intestinal barrier by enhancing tight junction protein expression, activating SCFA-sensing receptors (FFAR2/FFAR3), and suppressing NF-κB–mediated inflammation.^65^ ^,^ ^66^ In addition, acetate can promote mucus production and support epithelial metabolic demands during injury, limiting microbial translocation and barrier breakdown.^67^ Thus, we postulate that the UroA-associated increase in acetate may represent a key microbiota-dependent mechanism contributing to the restoration of gut barrier function and protection against iAs-induced intestinal damage observed in this study. Interestingly, we did not observe a significant alteration in SCFA levels in iAs-exposed mice compared with controls. This may be attributable to the limited number of control animals and the presence of outliers, which could have reduced the statistical power of the analysis. Additional studies with larger sample sizes will be required to more robustly assess the changes in SCFA levels and achieve statistical significance.

**Figure 8.**
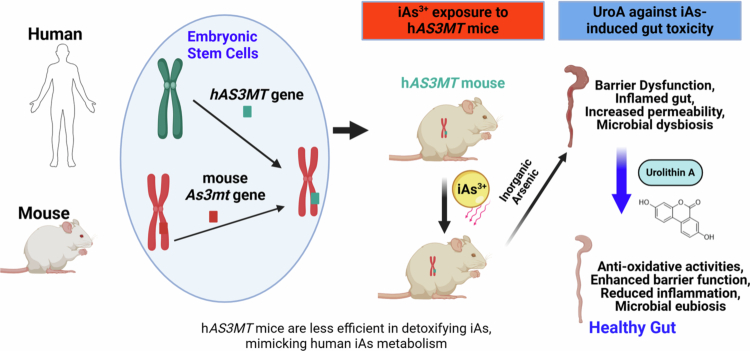
Experimental schematic illustrating the humanized AS3MT mouse model and the proposed protective effects of Urolithin A against arsenic-induced gut dysfunction. Mouse *As3mt* gene is replaced with human *AS3MT* gene and generated hAS3MT humanized mice. These mice exhibit reduced efficiency in iAs detoxification and more closely mimic human arsenic metabolism. The mice were exposed to iAs and treated with or without UroA. iAs exposure led to intestinal barrier dysfunction, gut inflammation, increased intestinal permeability, and microbial dysbiosis. Administration of Urolithin A (UroA) promoted antioxidative activity, enhanced intestinal barrier function, reduced inflammation, and restoration of microbial eubiosis, ultimately supporting the maintenance of a healthy gut.

Collectively, these findings reveal that UroA provides multifaceted protection against chronic iAs toxicity by strengthening the intestinal barrier, reducing oxidative stress and inflammation, and restoring microbiome balance. Given the global burden of arsenic exposure and the lack of effective therapies targeting intestinal injury and inflammation, UroA represents a promising dietary metabolite with translational potential (Figure 8).

Limitations of this study include the absence of an assessment of arsenic metabolism. It is plausible that UroA influences inorganic arsenic (iAs) methylation, which could partially explain the observed phenotypes. Additionally, the study did not stratify outcomes by sex, which may provide further mechanistic insights. While both male and female mice were included in this study, no significant sex-dependent differences in phenotype were observed. However, detailed sex-based analysis was not performed, and the estrous cycle of female mice was not assessed, which may contribute to variability in immune and microbiota-related outcomes. These limitations have been acknowledged and should be considered when interpreting the findings. To assess chronic effects, mice were exposed to 100 ppb inorganic arsenic (iAs) for 28 weeks to examine gut epithelial tight junction disruption and systemic impacts. For targeted evaluation of gut barrier injury with clinical relevance, a higher dose (400 ppb) over a shorter duration was used, based on pilot studies showing robust barrier dysfunction after 10 weeks; this regimen was therefore adopted for final experiments to assess UroA efficacy. We acknowledge the limitation that dose- and time-dependent effects of iAs exposure, as well as optimization of UroA treatment, remain to be determined and will be addressed in future studies. Interestingly, UroA exposure was associated with lower urinary arsenic levels compared to vehicle controls, although this difference did not reach statistical significance. The underlying mechanisms for this observation remain unclear. One possibility is that UroA alters arsenic bioavailability or facilitates its conversion into less harmful arsenic speciation forms, thereby mitigating tissue damage. An analysis of arsenic speciation (MMA vs. DMA) in iAs-exposed mice after UroA treatment would have been informative in clarifying the possible role of UroA in modulating iAs metabolism. Future studies will explore the molecular mechanisms underlying UroA's protective effects, including its influence on iAs metabolism (As speciation), mitochondrial function, SCFA signaling, and immune-microbiota interactions. Additionally, investigating UroA‑mediated increases in SCFAs (e.g., acetate and butyrate) and their downstream signaling through G‑protein–coupled receptors (GPR43, GPR41, and GPR109A) may elucidate novel crosstalk mechanisms between microbial metabolites and host physiology. We acknowledge that although UroA treatment significantly altered gut microbial composition, the current study does not provide functional validation of the specific bacterial taxa or microbial groups altered by UroA that may underlie its protective effects. Future studies will be necessary to establish a causal relationship between the microbiota and the observed phenotype. In particular, fecal microbiota transplantation experiments could be used to determine whether the protective effects are transferable through the gut microbiota, while antibiotic-mediated microbiota depletion approaches may help define the extent to which these effects depend on microbial communities. Together, such approaches would provide important mechanistic insights into UroA-mediated host–microbe interactions. Clinical trials will be essential to determine whether UroA supplementation can confer similar benefits in populations at risk for environmental arsenic exposure. This work advances our understanding of the interplay between iAs, microbial metabolites, and gut barrier function and opens avenues for investigating their broader impact on arsenic-induced multi-organ damage, including links to metabolic, neurological, and oncogenic disorders. Ultimately, UroA may offer a novel strategy for preventing and treating iAs-induced gut injury and related pathologies.

## Supplementary Material

Supplementary Figure 4.TIF Supplementary Figure 4.TIF

Supplementary Table 3.xlsx Supplementary Table 3.xlsx

Supplementary Table 4.xlsx Supplementary Table 4.xlsx

Supplementary Figure 3.TIF Supplementary Figure 3.TIF

Supplementary Figure 1.TIF Supplementary Figure 1.TIF

Supplementary Figure 2.TIF Supplementary Figure 2.TIF

Supplementary Table 5.xlsx Supplementary Table 5.xlsx

Supplementary Table 1 and Table 2.docx Supplementary Table 1 and Table 2.docx

## Data Availability

The raw reads of all 16S amplicons were deposited in the NCBI Sequence Read Archive (SRA) with the Bioproject ID: PRJNA1399987.
