# Supplementary figures and images for: Microbial metabolite Urolithin A protects against inorganic arsenic-induced gut barrier dysfunction in humanized AS3MT mice
Source: Gut Microbes. 2026 Jul 3;18(1):2696618. doi: 10.1080/19490976.2026.2696618 (PMC13348994; doi:10.1080/19490976.2026.2696618)

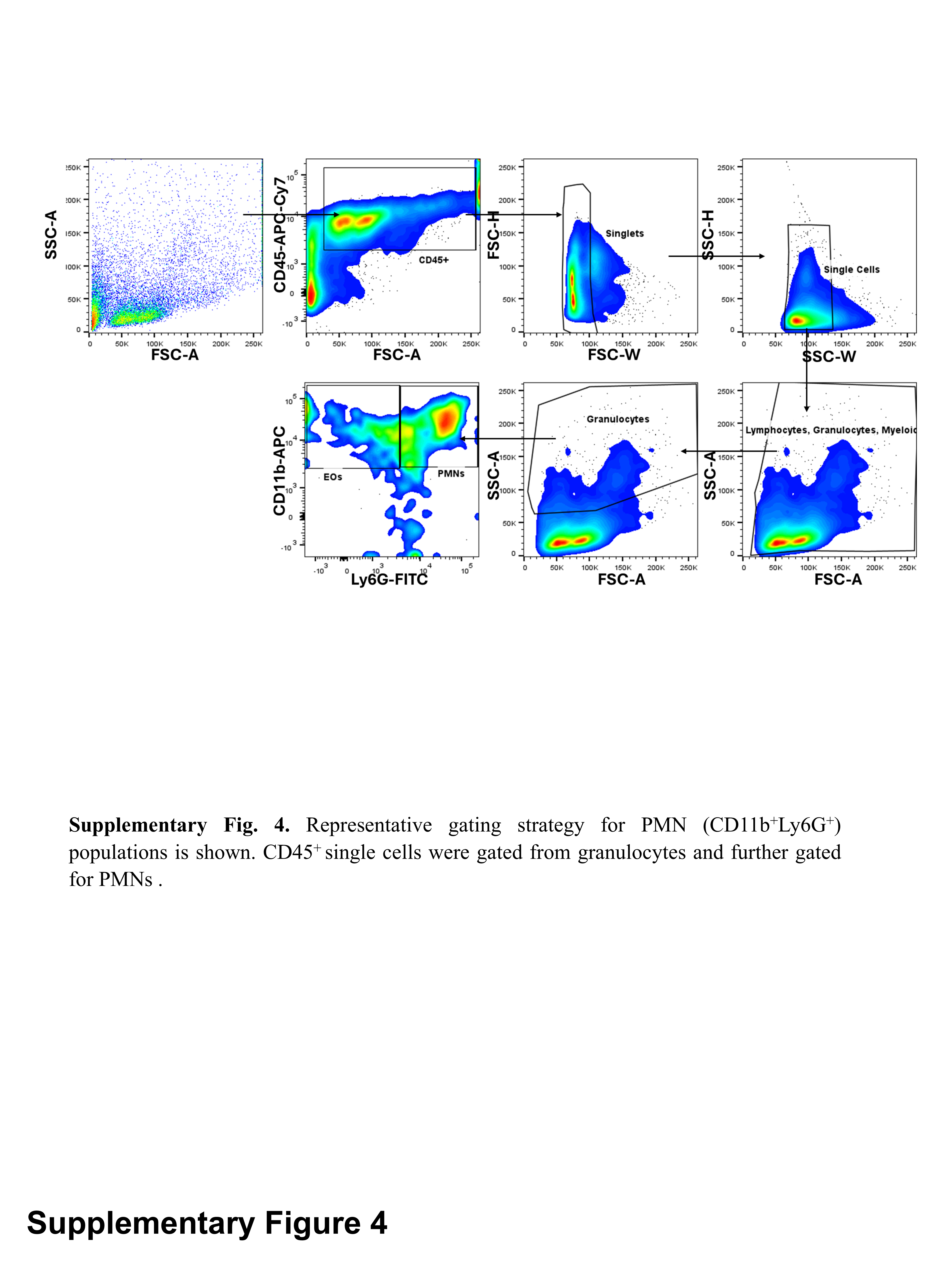

Supplement: Supplementary Figure 4.TIF [file KGMI_A_2696618_SM5992.tif]

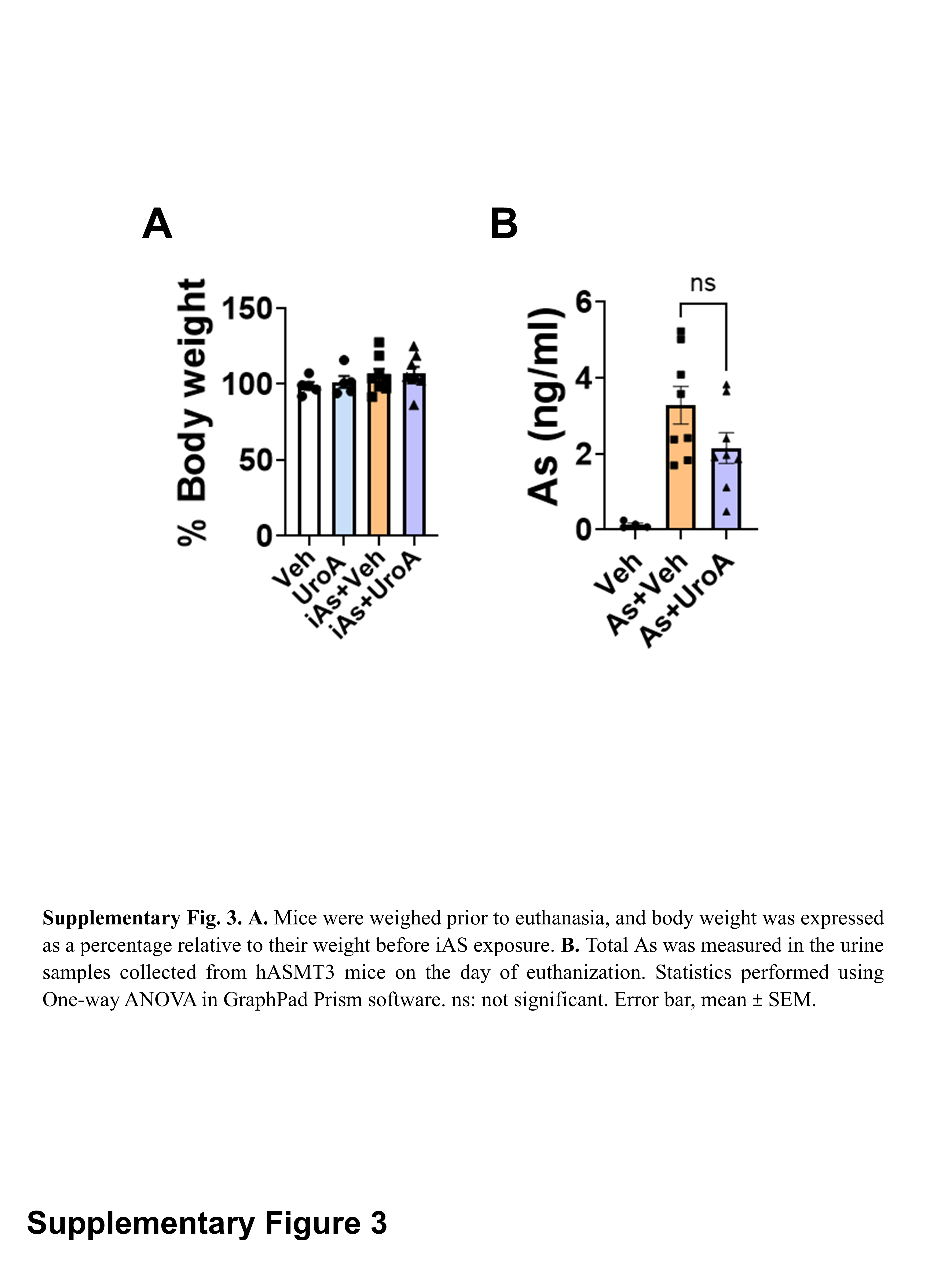

Supplement: Supplementary Figure 3.TIF [file KGMI_A_2696618_SM5995.tif]

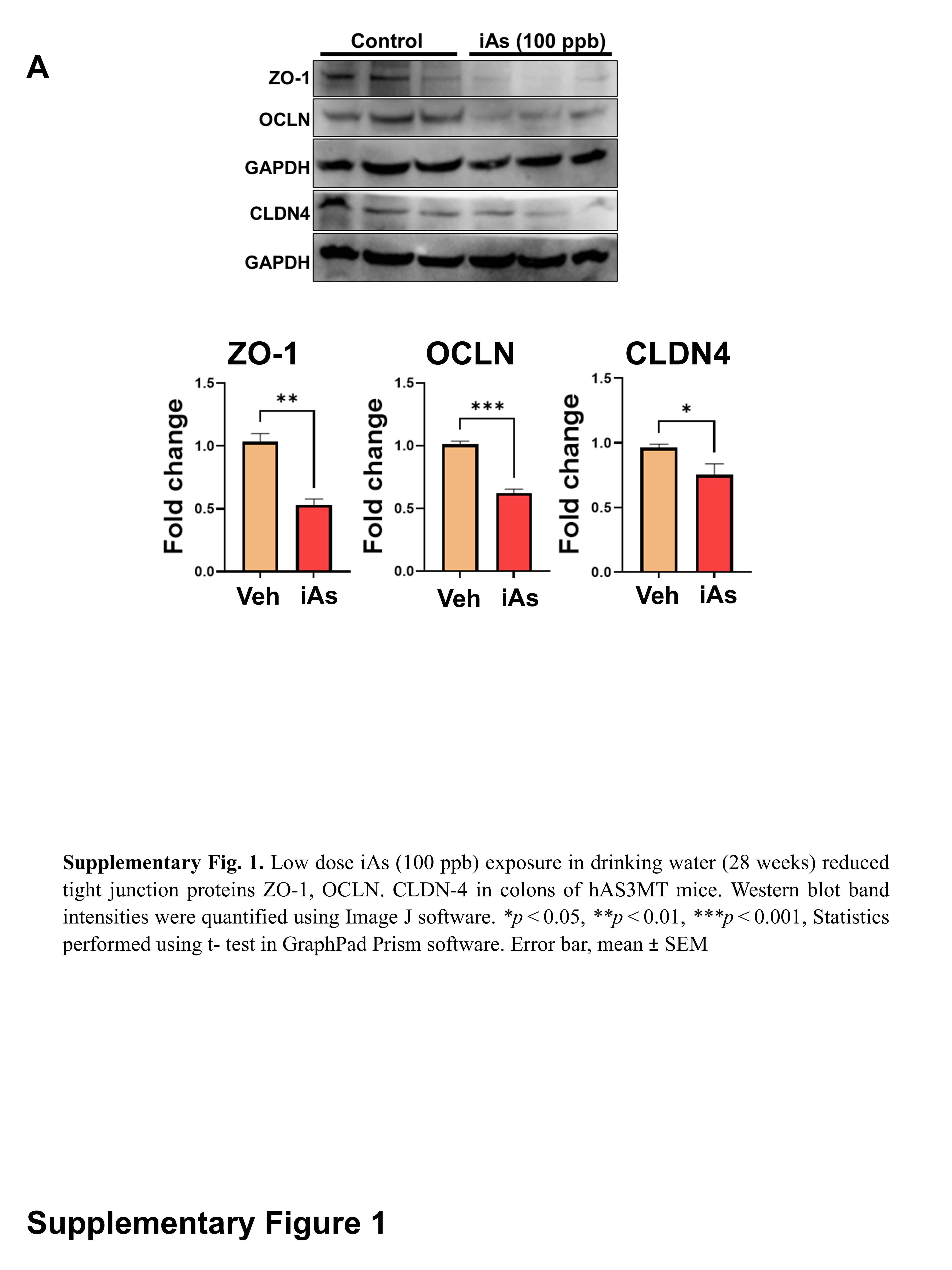

Supplement: Supplementary Figure 1.TIF [file KGMI_A_2696618_SM5996.tif]

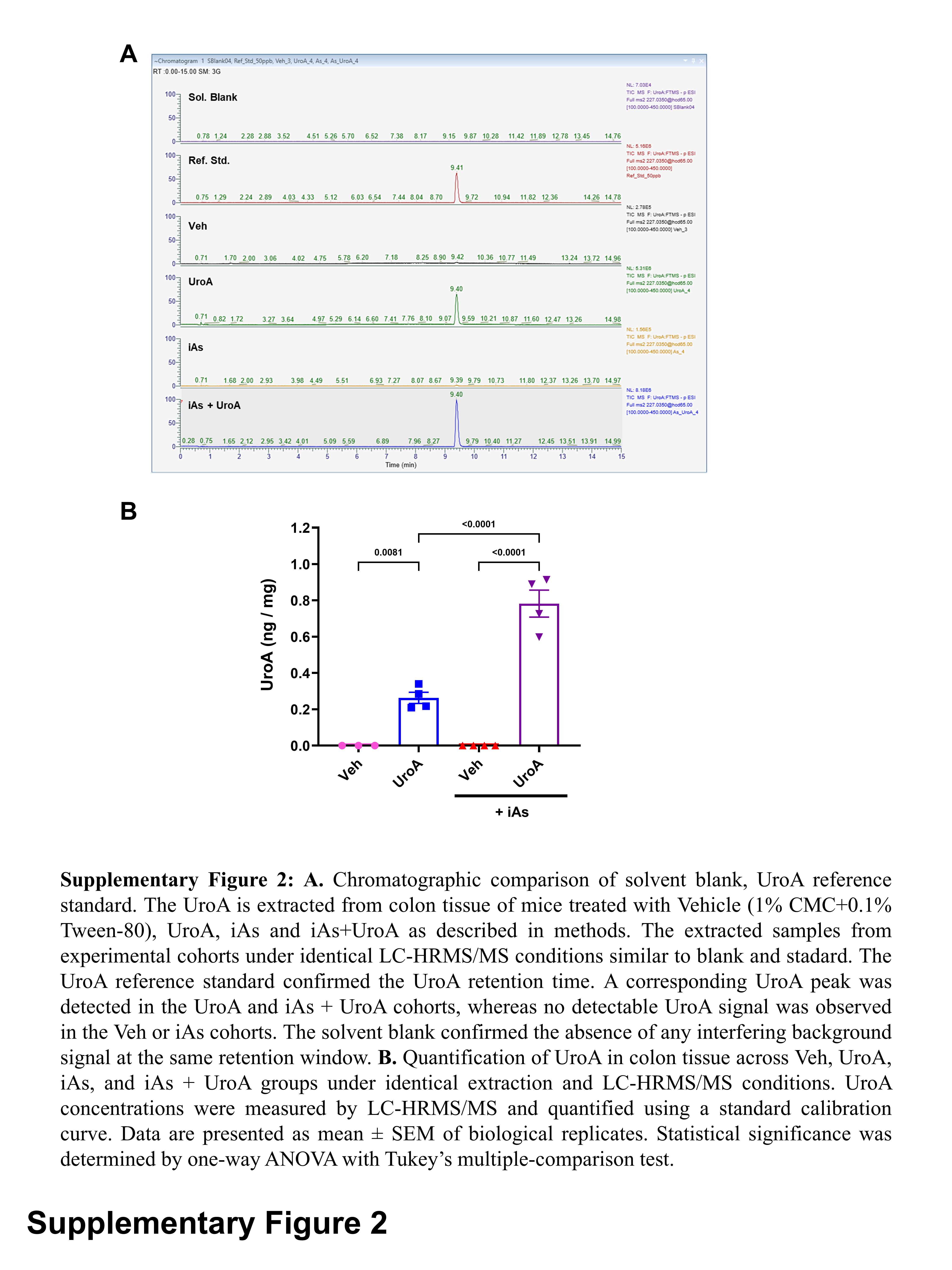

Supplement: Supplementary Figure 2.TIF [file KGMI_A_2696618_SM5999.tif]
