# Supplementary material for: Microbial metabolite Urolithin A protects against inorganic arsenic-induced gut barrier dysfunction in humanized AS3MT mice
Source: Gut Microbes. 2026 Jul 3;18(1):2696618. doi: 10.1080/19490976.2026.2696618 (PMC13348994; doi:10.1080/19490976.2026.2696618)
Supplement: Supplementary Table 1 and Table 2.docx [file KGMI_A_2696618_SM5998.docx]

**Supplementary Table 1:** List of the antibodies used in western blotting -

| **Antibody** | **Company** | **Catalog number** | **Dilution** |
| --- | --- | --- | --- |
| ZO-1 Antibody | ProteinTech | A21773-1-AP | 1:1000 |
| Claudin-4 Antibody (A-12) HRP | SCBT | sc-376643 HRP | 1:500 |
| Occludin Rabbit Polyclonal antibody | ProteinTech | 3409-1-AP | 1:1000 |
| Goat anti-rabbit IgG (H+L), HRP conjugate | ProteinTech | SA00001-2 | 1:5000 |
| HRP-Conjugated GAPDH | ProteinTech | HRP-60004 | 1:5000 |
| HRP-Conjugated Beta Actin | ProteinTech | HRP-60008 | 1:5000 |

**Supplementary Table 2:** List of antibodies used in flow cytometry -

| **Antibody** | **Company** | **Catalogue number** |
| --- | --- | --- |
| APC anti-mouse/human CD11b Antibody | BioLegend | 101211 |
| FITC anti-mouse Ly-6G Antibody | BioLegend | 127605 |
| APC/Cyanine7 anti-mouse CD45 Antibody | BioLegend | 103115 |
